# Supplementary material for: Causal interplay between lactose intolerance and gut microbiota: a combined bidirectional Mendelian randomization and in vivo validation study
Source: Front Nutr. 2026 Jun 1;13:1803337. doi: 10.3389/fnut.2026.1803337 (PMC13265576; doi:10.3389/fnut.2026.1803337)
Supplement: Supplementary file 1 [file Data_Sheet_1.zip › supplementary materials/Forward/leave one out cross validation/ebi-a-GCST90027531.finngen_R12_E4_LACTONAS.pdf]

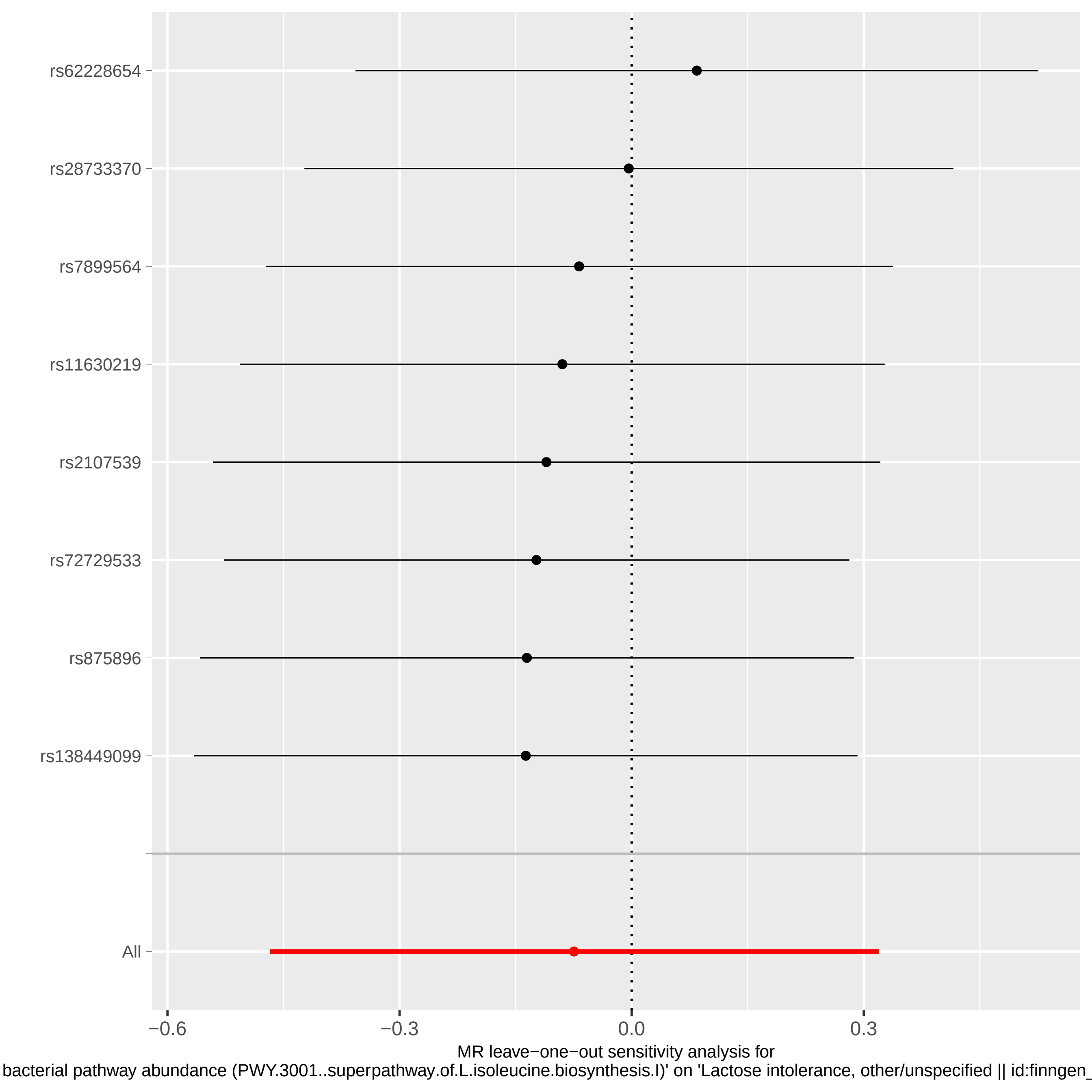
rs62228654
rs28733370
rs7899564
rs11630219
rs2107539
rs72729533
rs875896
rs138449099
All
−0.6
−0.3
0.0
0.3
MR leave−one−out sensitivity analysis for
bacterial pathway abundance (PWY.3001..superpathway.of.L.isoleucine.biosynthesis.I)' on 'Lactose intolerance, other/unspecified || id:finngen_
